# Importance of continuous monitoring of bovine leukaemia virus infection on farms in bovine leukaemia virus‐endemic areas of Japan

**DOI:** 10.1002/vro2.70014

**Published:** 2025-06-27

**Authors:** Chikako Tani, Jiazhou Li, Hirohisa Mekata, Kazuhiro Morishita, Shingo Nakahata

**Affiliations:** ^1^ Division of Tumor and Cellular Biochemistry Department of Medical Sciences University of Miyazaki Kiyotake Miyazaki Japan; ^2^ Division of HTLV‐1/ATL Carcinogenesis and Therapeutics Joint Research Center for Human Retrovirus Infection Kagoshima University Kagoshima‐shi Kagoshima Japan; ^3^ Center for Animal Disease Control University of Miyazaki Miyazaki Japan; ^4^ Division of Pediatrics Department of Developmental and Urological‐Reproductive Medicine Faculty of Medicine University of Miyazaki Kiyotake Miyazaki Japan

## Abstract

**Background:**

Enzootic bovine leukosis (EBL), caused by the bovine leukaemia virus (BLV), has exhibited increasing infection rates in Japan. While many farms implement various infection prevention measures, practical challenges—such as barn structure and availability of replacement cattle—can hinder farm purification efforts. This study aimed to determine whether continuous monitoring of BLV proviral load (PVL) in cattle on a farm could be effective in tracking BLV infection dynamics, identification of BLV infection sources and the dynamics of BLV PVL enrichment on‐farm.

**Methods:**

Bovine leukaemia virus infection was monitored over a 3‐year period in approximately 10% of cattle at a test farm located in Miyazaki Prefecture, an endemic area for BLV in southern Kyushu, Japan. Eight calves and five adult cows were included to assess vertical and horizontal transmission of BLV.

**Results:**

All calves developed new BLV infections, which were traced to the nursing/growing barn, the dry/new milking barn at the Miyazaki farm, and the point of deposit in Hokkaido, Japan, for rearing and artificial insemination. In BLV‐positive adult cows, an increasing trend in PVL was observed. These findings indicate that continuous monitoring of a subset of cattle enables effective tracking of infection dynamics and identification of infection sources; thus, supporting the implementation of targeted prevention measures.

**Conclusions and clinical importance:**

Despite heightened awareness among farmers regarding livestock quarantine, consistent and long‐term control of BLV infections remains challenging. The results underscore the necessity of expert‐guided management strategies tailored to BLV control, informed by ongoing infection monitoring. This study highlights husbandry management factors, including barn structure and cattle boarding practices, and provides new insights into the transmission routes of BLV in Japan.

## INTRODUCTION

Enzootic bovine leukosis (EBL) was first reported in Denmark in the 1960s,^1^ and is currently widespread worldwide, except in EBL‐free countries.^2^, ^3^, ^4^, ^5^ Enzootic bovine leukosis is caused by bovine leukaemia virus (BLV), a delta retrovirus closely related to human T‐lymphotropic virus type 1 (HTLV‐1).^6^ According to the Japanese policy, all cattle that develop EBL have to be slaughtered, and any products such as milk and meat obtained from these cattle must be discarded, resulting in significant losses for farmers. Some infected cattle, even if they do not show any symptoms, may develop persistent lymphocytosis (PL).^7^ Persistent lymphocytosis cattle have been reported to develop mastitis due to a weakened immune system,^8^ which has a negative impact on the economy due to reduced productivity.^4^, ^5^, ^9^

In Japan, the number of cases for EBL have been increasing since its inclusion as a notifiable infectious disease in 1998.^10^, ^11^ Antibody tests for BLV in six cattle herds in Hokkaido during 1977–1979 showed a rapid increase in the positivity rate, from 18.1% in 1977 to 38.0% in 1978 and 64.5% in 1979.^12^ In 1998, EBL was designated a notifiable disease; however, the antibody tests conducted in Japan from 2009 to 2011 demonstrated a BLV positivity rate of 40.9% in dairy cows and 28.7% in beef cattle, a 10‐fold increase in dairy cows and a four‐fold increase in beef cattle compared to the results of tests conducted in 1980–1982.^10^ The Ministry of Agriculture, Forestry, and Fisheries of Japan issued the ‘Guideline for Hygienic Measures for Enzootic Bovine Leukosis’, which introduced control measures for farms and related parties, such as measures during parturition, against blood‐sucking insects to prevent horizontal infection, and for the placement of cows on farms.^13^ In particular, the guidelines state that ‘BLV‐infected cows should be separated from non‐infected cows at the time of calving, and once an infected cow has given birth, the calf should be immediately separated and raised separately. Furthermore, colostrum must come from a non‐infected cow, or colostrum from a BLV‐infected cow should be frozen and then thawed before being fed’. However, measures to prevent EBL have not been effective, and the number of infected cattle has been rapidly increasing year by year since 1999.^13^

In Japan, antibody tests are commonly used to diagnose BLV infections. However, it is extremely important to measure the proviral load (PVL) of BLV using polymerase chain reaction (PCR) tests.^14^, ^15^, ^16^ Attempts are being made to use PVL as an indicator to cull or isolate highly infectious cows.^14^, ^15^ In addition, PVL in cows is used as an indicator to prevent mother‐to‐child infection.^16^ Therefore, PVL measurement is important for the accurate diagnosis of BLV. High PVL levels are an important risk factor for progression to EBL and may be used as an indicator to identify cows that should be culled from the herd long before the development of EBL.^17^ Furthermore, for economic reasons, many clinical sites, including veterinary clinics and agricultural mutual aid associations, in Japan do not perform antibody or PCR tests. Therefore, the diagnosis of EBL is based on clinical findings and general blood tests, mainly focusing on lymphocyte counts.^18^ The ‘European Community's leukosis (EC) key’, which uses blood lymphocyte counts as an indicator, has long been recognised as a screening test for BLV.^1^ In Japan, antibody tests for BLV infection are performed by livestock hygiene centres; however, owing to the cost and lack of personnel, they can only test a portion of cattle, and general veterinary hospitals and NOSAI (Agricultural Mutual Aid Association) mainly diagnose EBL based on the lymphocyte count. However, the normal range of lymphocyte counts varies based on the breed. Moreover, studies have reported EBL types with no increase in lymphocyte counts^19^, ^20^; thus, clinically diagnosing BLV‐infected cattle based on lymphocyte counts is challenging.

This study aimed to clarify whether continuous monitoring of BLV PVL in cows on a farm could be useful in tracking BLV infection dynamics, identifying BLV infection sources, and elucidating the dynamics of BLV PVL enrichment in farms.

## METHODS

### Herd demographics and husbandry

The test farm is located in Miyazaki Prefecture, Japan. At the beginning of the study, approximately 120 Holstein dairy cows were housed in a free‐stall barn, where adult cows milked in the milking parlour were fed a total mixed‐ration diet. The old and new barns of the farm are described below.

**FIGURE 1:**
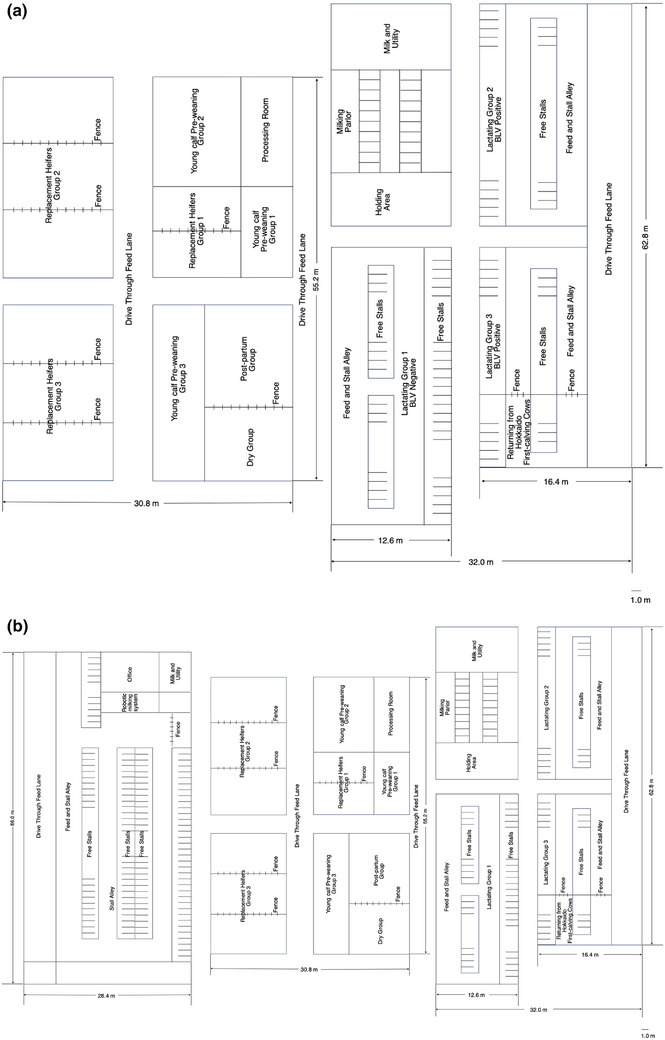
Comparison of old and new barn layouts. (a) Old barn. (b) New barn. The calf and heifer barns had undergone multiple renovations and expansions, resulting in all barn roofs—including that of the former milking barn—being interconnected. In contrast, the new barn was a separate structure, newly constructed adjacent to the existing facilities. The old barn was a naturally ventilated structure equipped with fans, whereas the new barn was a cross‐ventilated robotic milking barn.

Figure 1a illustrates the layout of the old barn. Lactating cows were categorised into Group 1 (BLV negative) and Groups 2 and 3 (BLV positive). Upon completing lactation, the cows were transferred to the dry cow group. These cows gave birth to calves approximately 2 months later and were subsequently placed into the post‐partum group for about 1 week. On this farm, both dry cow and post‐partum groups were not separated into BLV‐positive and ‐negative groups due to space limitations. They were then returned to the lactation group for milking. Newborn calves (approximately 80) were separated from their dams immediately after birth and housed in individual pens or calf hutches (young calf pre‐weaning Groups 1, 2 and 3), where they consumed colostrum. Subsequently, they were fed with milk replacer and starter feed for approximately 40 days. After weaning, the calves were moved to group loose pens accommodating five to seven animals (replacement heifers Groups 1, 2 and 3) and were fed a growth diet.

Figure 1b illustrates the layout of the new barn. Milking of cows was managed using a robotic milking system. Upon completing lactation, the cows underwent the dry period and calving (transition period) in the old barn before returning to the new barn for milking. Calves were reared similarly in the old barn. To prevent BLV infection, the colostrum fed to the calves on this farm was milked from another cow, frozen and thawed before feeding.^21^ At the old barn, the colostrum given to the calves was initially frozen from colostrum from negative cows and then thawed just before feeding. However, after moving to the new barn, it became impossible to manage positive and negative cows separately; hence, colostrum from both negative and positive cows was processed in the same way and fed to the calves. In addition, calves and heifers were tested irregularly for their BLV status; however, owing to the size and structure of the facility, they were not kept separately. At this farm, calves are handled exclusively by specific personnel, and after weaning, all the farm staff handle the calves. The farm had taken measures to prevent various infectious diseases, including BLV, such as changing gloves, boots and clothing for each cow and herd, and installing a disinfectant tank at the entrance of each cow shed.

At the beginning of the study, as a preventive measure against BLV infection, adult cows in two free‐stall barns (old barns) housing approximately 60 cows were labelled with different colours to distinguish between BLV‐positive and BLV‐negative cows (Figure 1a). Bovine leukaemia virus‐positive and ‐negative cows were kept separately in their respective barns. One year later, a milking robot was introduced, and a free‐stall milking barn housing approximately 120 cows (new barn) was built (Figure 1b). Additionally, 120 new adult cows were added, increasing the total number of cows to approximately 240. The 120 cows introduced into the free‐stall milking barn were not tested for BLV antibodies, and their BLV infection status was unknown. The layout of the entire barn was significantly changed, with cows eligible for robotic milking relocated to the new milking barn (new barn robotic milking system). Cows that could not be milked by robots were placed in the old barn (lactating group), and as a result, it became impossible to separate the BLV‐positive and BLV‐negative cows (Figure 1b). As this is an observational pilot study, and the general guideline is to use a sample size of 10% of the main study,^22^ in Study A, eight calves were randomly selected from 80 newborn calves and used for the test. Similarly, in Study B, approximately 50 BLV‐infected cows were kept in the positive barn at the start of the study, and five of them were randomly selected for monitoring. These five adult cows were housed in a separate area from the calves in Study A. The calves produced from these cows were not included in Study A. Additionally, the calves were separated immediately after calving; thus, they had no contact with their dams and did not receive colostrum directly from their dams' udders.

### Laboratory testing

Eight calves (10.4 ± 6.7 days old) were included in Study A. Blood samples were collected from the jugular vein using a disposable syringe and stored in heparinised blood collection tubes with a storage capacity of 10 mL (Venoject II, Terumo) at the start of the study and approximately 2, 5, 8, 22 (immediately after returning to Miyazaki from Hokkaido, where they were entrusted for 1 year) and 36 months (1 year after returning to Miyazaki) for observing clinical symptoms, including visual and palpatory examination to check for any abnormalities in vitality, energy, stool condition and superficial lymph nodes.

Five adult cows (3.1 ± 0.5 years old) positive for BLV were used in Study B. Blood samples were collected from cows in the positive barn (Figure 1a) at the start of the study and at 2, 5 and 12 months to observe clinical symptoms. The samples were brought to the laboratory at room temperature. The white blood cells and lymphocytes were measured using a fully automated blood cell counter, MEK‐6550 Cell‐Tac α (Nihon Kohden), as per the manufacturer's instructions.

**FIGURE 2:**
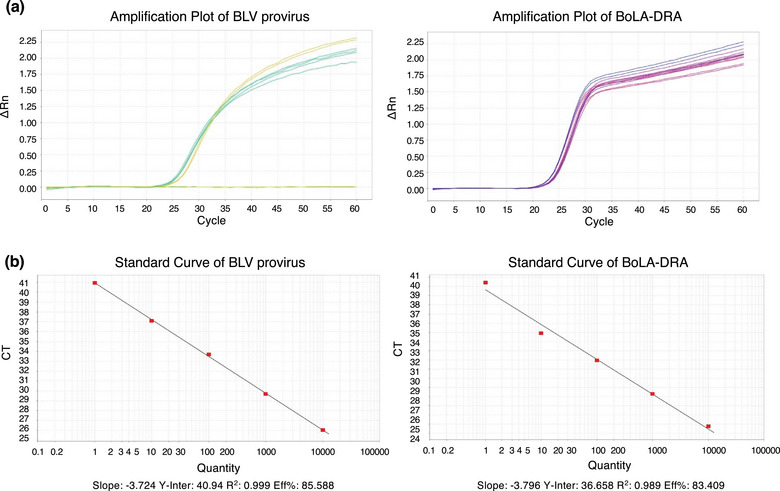
Representative amplification plots (a) and standard curves (b) from real‐time PCR analysis of bovine leukaemia virus provirus and BoLA‐DRA. Data are from Calve Nos. 2, 3 and 4 at 5 months and Cow Nos. 1, 3 and 4 at 12 months.

Peripheral blood mononuclear cells were isolated from whole blood using Histopaque‐1077 (Sigma‐Aldrich), and genomic DNA was extracted. The PVL of BLV was measured using the StepOnePlus real‐time PCR system (Thermo Fisher Scientific) with a Coordination of Common Motifs (CoCoMo)‐BLV Primer/Probe (Riken Genesis).^23^, ^24^, ^25^ The BLV‐CoCoMo‐qPCR method targets the BLV LTR region and amplifies and detects both the integrated BLV provirus and the host gene (BoLA‐DRA) in real‐time PCR. The PVL quantification method using BLV‐CoCoMo‐qPCR employs a reduced primer set designed using the CoCoMo algorithm.^23^ This allows for amplification of nearly all BLV strains, including unknown ones. Simultaneous detection of the host BoLA‐DRA gene enables accurate calculation of the copy number per cell. The sensitivity and reproducibility of BLV‐CoCoMo‐qPCR are superior to two previously developed real‐time PCR methods (i.e., the TaqMan MGB assay and the TaKaRa cycleave PCR).^24^ Furthermore, the sensitivity of BLV‐CoCoMo‐qPCR is 1.9 times greater than that of nested PCR, with the ability to detect PVLs ranging from zero to five copies per 10^5^ cells.^23^, ^24^ PCR amplification was performed in a 20‐µL reaction mixture containing 1× TaqMan Gene Expression Master Mix, BLV primer, FAM‐BLV probe, and 30 ng of template DNA. Additionally, the BoLA‐DRA region was amplified (Figure 2). The PVL was calculated as follows:

BLV PVL = [BLV provirus copy number/diploid cell number] × 100,000 cells = [(BLV LTR copy number/2)/(BoLA‐DRA copy number/2)] × 100,000 cells.^23^

## RESULTS

In Study A, none of the eight calves had PVL until 2 months of age, indicating that there was no vertical infection. By 22 months of age, 37.5% (3/8) exhibited temporary lymphocyte counts exceeding 12,000/µL—a threshold value for calves in the EC key (Table 1). Calve Nos. 5 and 7 had lymphocyte counts of 12,400/µL and 13,600/µL at 8 months of age, respectively. However, their PVL was 1 copy/100,000 cells and 0 copy/100,000 cells, suggesting that PVL might not be directly related to the increase in lymphocyte count (Table 2). On the other hand, the lymphocyte count of Calf No. 1 was 12,000/µL at 22 months of age, exceeding the EC standard, and the PVL was also 1363 copies at that time (Table 2), suggesting that the lymphocyte count may have increased in conjunction with BLV infection.

**TABLE 1:** Time course of peripheral blood lymphocyte counts in calves in Study A.

Calf No.	Within 25 days	2 months	5 months	8 months	22 months	36 months
1	6000	8800	9200	8400	12,000	ND
2	6000	9600	8000	5200	6400	ND
3	2800	3600	8800	7600	10,000	ND
4	4400	7600	6000	3600	4800	ND
5	7200	5600	8000	12,400	6400	ND
6	10,400	11,200	11,600	9600	5200	ND
7	7600	10,800	8400	13,600	6800	ND
8	7600	10,000	10,000	4000	ND	ND

*Note*: Peripheral blood lymphocyte counts are shown in units of cells per microlitre of blood (cells/µL). The 22‐month‐old age indicates the age immediately after returning to Miyazaki Prefecture after 1 year of fostering in Hokkaido.

Abbreviation: ND, not determined.

**TABLE 2:** Time course of bovine leukaemia virus proviral loads in calves in Study A.

Calf No.	Within 25 days	2 months	5 months	8 months	22 months	36 months
1	0	0	0	0	1363	Disuse
2	ND	0	0	0	0	Disuse
3	0	0	0	0	33,933	ND
4	0	0	0	3	27	28
5	0	0	0	1	7	383
6	0	0	0	0	0	8
7	0	0	0	0	0	37,388
8	0	0	ND	0	0	44,881

*Note*: Proviral loads are shown in units of copies per 10^5^ peripheral blood mononuclear cells. Bovine leukaemia virus‐CoCoMo‐qPCR measured the proviral load in blood.^23^ The 22‐month‐old age indicates the age immediately after returning to Miyazaki Prefecture after 1 year of fostering in Hokkaido, and the 36‐month‐old age indicates the age 1 year after returning to Miyazaki Prefecture. Calves Nos. 1 and 2 were shipped to the slaughterhouse at 36 months of age (Disuse).

Abbreviation: ND, not determined.

Two calves tested positive for BLV by 8 months of age: No. 4 had a PVL of 3 copies/100,000 cells, and No. 5 had 1 copy/100,000 cells (Table 2). These two calves continued to test positive until the age of 36 months. Therefore, it was suggested that Nos. 4 and 5 might have been horizontally infected on this farm by the age of 12 months. In contrast, Nos. 1 and 3 were BLV‐negative at 8 months of age. However, upon their return from Hokkaido to Miyazaki Prefecture, they exhibited PVL counts of 1363 copies/100,000 cells and 33,933 copies/100,000 cells, respectively. This suggested that Nos. 1 and 3 were likely infected horizontally during the deposition period in Hokkaido. Furthermore, at 36 months of age, 1 year after returning from the deposit in Hokkaido to the Miyazaki Prefecture farm, three cows (No. 6: 8 copies/100,000 cells; No. 7: 37,388 copies/100,000 cells; and No. 8: 44,881 copies/100,000 cells) tested positive for BLV. These BLV‐positive cases were suggested to have been caused by a horizontal infection at the Miyazaki Prefecture farm. Furthermore, these BLV infections were identified to have occurred in the nursing/growing barn and the dry/new milking barn of the test farm (Table 3, Figure 1), except for the time of deposit in Hokkaido, and it was revealed that they occurred throughout almost the entire farm.

**TABLE 3:** Estimated route of bovine leukaemia virus infection in Study A.

Place of infection	Cattle No.	Mode of infection	Age	Number of heads (%)
Replacement Heifers Group 3	4, 5	Horizontal	<1	2 (25)
Hokkaido	1, 3	Horizontal	1–2	2 (25)
Free stalls	6, 7, 8	Horizontal	>2	3 (37.5)

*Note*: No cases of placental or birth canal infection were estimated in Study A. Calf No. 2 was not infected with bovine leukaemia virus during the observation period and is therefore not shown in the table. Calves Nos. 4 and 5 were found to be newly infected in the Replacement Heifers Group 3 area of the old barn (Figure 1a). Calves Nos. 6, 7 and 8 were found to be newly infected in the free‐stalls area of the new barn (Figure 1b).

In Study B, involving five adult cows positive for BLV, 80% (four of five cows) had lymphocyte counts exceeding 8500/µL—a threshold value for adult cows defined in the EC key—at the start of the study (Table 4). By 12 months into the study, four cows (Nos. 2, 3, 4 and 5) had lymphocyte counts exceeding 8500/µL, with counts of 13,200, 8800, 9600 and 25,200/µL, respectively. The mean PVL was 39,334 ± 28,517 copies/100,000 cells at the start of the study, but by the end of the study, the cow's PVL was 59,207 ± 22,344 copies/100,000 cells (Table 5). Additionally, adult cows with high PVL were kept in a new milking barn, which may have been the source of BLV infection (Table 3, Figure 1). No cows developed lymphatic tumours during the study in either Study A or B.

**TABLE 4:** Time course of peripheral blood lymphocyte counts in cows in Study B.

Cow No.	0 month	2 months	5 months	12 months
1	4400	6000	3600	3200
2	15,600	16,800	14,800	13,200
3	9200	8400	8000	8800
4	10,400	12,400	9600	9600
5	19,600	20,400	20,400	25,200

*Note*: Proviral load counts are shown in units of cells per microlitre of blood (cells/µL). 0 month = at the start of the study. Throughout Study B, all subject cows were housed in a free‐stall area designated for the bovine leukaemia virus‐positive group within the old barn (Figure 1a).

**TABLE 5:** Time course of bovine leukaemia virus proviral loads in cows in Study B.

Cow No.	0 month	2 months	5 months	12 months
1	8538	11,117	10,491	27,317
2	36,067	54,275	36,844	82,572
3	15,102	19,130	20,606	45,644
4	66,580	59,064	42,279	71,998
5	70,385	38,609	76,399	68,506

*Note*: Proviral loads are shown in units of copies per 10^5^ peripheral blood mononuclear cells. Bovine leukaemia virus‐CoCoMo‐qPCR was used to measure the proviral load in blood.^23^ 0 month = at the start of the study. Throughout Study B, all subject cows were housed in a free‐stall area designated for the proviral load‐positive group within the old barn (Figure 1a).

## DISCUSSION

The results of Study A showed that the BLV infection rate over 3 years was 100% (seven of seven heads), excluding calf No. 2 (Table 2). Calf No. 2 remained negative until 22 months of age, but had already been shipped to the slaughterhouse by 36 months, so it is unknown whether it eventually became positive. The test cohort comprised eight heads, representing approximately 10% of the calves on the farm. Monitoring their infection status allowed for the identification of the mode and location of BLV infection within the herd. This suggests that for economic reasons, monitoring a subset of cattle can effectively reveal BLV infection status and routes of transmission, aiding infection control.

In the present study, we confirmed that infection occurred when calves were entrusted to Hokkaido for breeding purposes. In 1980, Onuma et al. recommended testing for BLV antibodies in cattle,^12^ and the Japanese ‘Guideline for Hygienic Measures for Enzootic Bovine Leukosis’ emphasises the need for such testing.^13^ The introduction and movement of cattle can lead to the spread of BLV infection and should be performed with caution. However, this recommendation has not been fully used in clinical practice in Japan. Additionally, entrusting cattle to other farms has traditionally been considered problematic in terms of disease prevention. Despite this, entrusting cattle to other farms has continued, as it is more economical than raising calves on their own farms in terms of the construction of breeding facilities, cost of feed for feeding management, and employment of managers. Although BLV testing in Japan before and after entrustment is not mandatory, it is considered essential for such entrustment and introduction. As awareness about BLV strains increases, the movement of cattle from diverse locations complicates infection control.^26^, ^27^ Additionally, the risk of introducing other infectious diseases necessitates in‐house breeding methods, which may become increasingly important.

After returning the cow from Hokkaido to the test farm, the presence of an infected cow was confirmed in a new barn. At the start of this study, cows were separated into two old barns based on whether they were infected with BLV, but the layout based on the presence or absence of BLV infection was not reflected in the new barn that was built during the study period. Therefore, BLV‐positive and BLV‐negative cows were mixed 24 hours a day, which is likely to have caused the spread of BLV infection. While there have been previous discussions on barn layout,^28^ few have addressed quarantine measures. Expert guidance on infectious disease prevention will be crucial when constructing new barns.

The BLV PVLs in cattle are considered to remain stable after the acute phase following BLV infection.^29^ However, changes in PVLs have also been reported to be positively associated with changes in lymphocyte counts in infected cattle.^30^, ^31^ Moreover, an increase in PVLs is associated with the progression of PL.^14^ The dynamics of PVL following infection are reportedly related to the differences in the immune status.^32^ Moreover, as most of the cattle in Study B had high PVLs and some of them showed PL pathology, it is speculated that the immune status may be related to the observed PVL fluctuations in this group. High PVL levels and ageing are important risk factors for progression to EBL.^17^ In Study B, the PVL of BLV‐infected cows was followed up for 1 year, and four of five cows demonstrated an increase in the PVL. Although no cows developed EBL during the study period, the number of cows developing EBL may increase on this farm in the future. Furthermore, high PVL in dams has been linked to placental and birth canal infections.^16^ Monitoring the PVL of these cows is essential to prevent transmission to calves, which can occur through these routes. Many of the adult cows in Study B had high PVL and transitioned to PL cows. It is also possible that BLV infection may become more prevalent on this farm in the future. Study A suggested that infection occurs in the nursing and rearing barns. Although no placentally infected calves were identified in this group, a PVL assessment of a neonatal calf born to a BLV‐infected dam on the same farm revealed findings suggestive of placental transmission. Specifically, a case was identified within the first week of life in which the calf exhibited a markedly high PVL of 19,994 copies per 10⁵ peripheral blood mononuclear cells, despite having a lymphocyte count within the normal range (3200/µL). It is presumed that such calves, infected in utero, may have contributed to the spread of BLV within the nursing and rearing barns. There is a close association between the age at which infection occurs and the location of infection (i.e., inside or outside the barn), and identifying these factors is considered crucial for implementing effective BLV control measures.

Even the farmers who are highly aware of BLV infection prevention measures are unable to implement them, as economic considerations often influence barn construction and cattle entrustment, potentially undermining existing measures. Barn layouts and BLV infection conditions vary among farms. Additionally, infection prevention strategies vary widely among farms, and recognising the challenges of controlling BLV spread is essential for long‐term success. To achieve this, it is necessary to continue conventional measures while understanding changes in farm configuration and BLV infection status and to explore individual measures.

The present study focused on husbandry management factors, such as barn structure and cattle boarding practices, and provided new insights into the understanding of BLV transmission routes in Japan. This study also suggests that ongoing monitoring of cows' PVL can lead to effective BLV infection prevention measures, with potential applications in livestock farming practices in the future.

## AUTHOR CONTRIBUTIONS

Chikako Tani collected samples and performed the clinical examination. Chikako Tani, Hirohisa Mekata and Shingo Nakahata acquired and analysed the data. Chikako Tani drafted the manuscript. Shingo Nakahata edited the manuscript. Jiazhou Li analysed the data. Kazuhiro Morishita analysed the data and edited the manuscript.

## CONFLICTS OF INTEREST

The authors declare they have no conflicts of interest.

## FUNDING INFORMATION

This research was partly supported by Japan Society for the Promotion of Science KAKENHI Grant Number 18K07203.

## ETHICS STATEMENT

This study was approved by the Animal Experiment Review Board of the University of Miyazaki, Japan. Standards related to the care and maintenance of industrial animals in Japan have been followed.

## Data Availability

The data that support the findings of this study are available from the corresponding author upon reasonable request.
